# *Cthulhu Macrofasciculumque* n. g., n. sp. and *Cthylla Microfasciculumque* n. g., n. sp., a Newly Identified Lineage of Parabasalian Termite Symbionts

**DOI:** 10.1371/journal.pone.0058509

**Published:** 2013-03-18

**Authors:** Erick R. James, Noriko Okamoto, Fabien Burki, Rudolf H. Scheffrahn, Patrick J. Keeling

**Affiliations:** 1 Department of Botany, Canadian Institute for Advanced Research, University of British Columbia, Vancouver, British Columbia, Canada; 2 University of Florida Research and Education Center, Davie, Florida, United States of America; J. Craig Venter Institute, United States of America

## Abstract

The parabasalian symbionts of lower termite hindgut communities are well-known for their large size and structural complexity. The most complex forms evolved multiple times independently from smaller and simpler flagellates, but we know little of the diversity of these small flagellates or their phylogenetic relationships to more complex lineages. To understand the true diversity of Parabasalia and how their unique cellular complexity arose, more data from smaller and simpler flagellates are needed. Here, we describe two new genera of small-to-intermediate size and complexity, represented by the type species *Cthulhu macrofasciculumque* and *Cthylla microfasciculumque* from *Prorhinotermes simplex* and *Reticulitermes virginicus*, respectively (both hosts confirmed by DNA barcoding). Both genera have a single anterior nucleus embeded in a robust protruding axostyle, and an anterior bundle flagella (and likely a single posterior flagellum) that emerge slightly subanteriorly and have a distinctive beat pattern. *Cthulhu* is relatively large and has a distinctive bundle of over 20 flagella whereas *Cthylla* is smaller, has only 5 anterior flagella and closely resembles several other parababsalian genera. Molecular phylogenies based on small subunit ribosomal RNA (SSU rRNA) show both genera are related to previously unidentified environmental sequences from other termites (possibly from members of the Tricercomitidae), which all branch as sisters to the Hexamastigitae. Altogether, *Cthulhu* likely represents another independent origin of relatively high cellular complexity within parabasalia, and points to the need for molecular characterization of other key taxa, such as *Tricercomitus*.

## Introduction

The hindgut of lower termites has long been known for the diversity of its community of symbiotic protists [1]. The community is dominated by Parabasalia, which are not only of interest due to the critical ecological role they play in the breakdown of lignocellulose, but also because these symbionts have evolved a remarkable range of sizes and structural complexities [2]. The largest and most complex so-called hypermastigotes can reach hundreds of microns in length (visible to the naked eye) and can be covered by as many as 50,000 flagella, underpinned by a highly organized suite of cytoskeletal elements [2]–[7]. At the other end of the extreme are many other species of small, simple flagellates, typically with three to six flagella, and most likely similar to the common ancestor of the parabasalians as a whole [2], [8].

The large and complex hypermastigotes are most conspicuous and have therefore attracted the most attention in studies of the diversity and taxonomy of termite symbionts. Indeed, after over 100 years of study, it is arguable that most of the major types (i.e., genera) of large and complex hypermastigotes have probably been observed and described (although it is also increasingly clear that most species, even of the large parabasalian genera, remain to be described). The likelihood that many, if not most, of the major types of large hypermastigtes have been described is also supported by phylogenetic analysis of molecular surveys of hindgut diversity: there are many lineages in the tree of parabasalians that are made up entirely of “unidentified environmental sequences” from termite hindguts [9], but only a few of these are likely to represent organisms of large size and substantial complexity. Thus, current data suggest that most of the unexplored diversity of parabasalians will correspond to relatively small and simple flagellates, which will make up the bulk of the molecular diversity of the group.

In order to fully understand the evolution of the large, complex, and conspicuous parabasalians, it is necessary to pay greater attention to the smaller forms because the phylogeny of parabasalians makes it clear that large size and structural complexity have evolved more than once [2], [9]–[13]. The basic cytoskeletal unit of parabasalian body plans is the karyomastigont, a suite of connected structures including the nucleus, basal bodies and flagella, and accessory cytoskeletal and endomembrane structures [2], [14]. In some cases, cell size has increased but the karyomastigont complexity does not [11], [15]. In other cases, cellular complexity has increased by massive duplication of the entire karyomastigont system resulting in huge multinucleate cells [13], [16]–[18]. In still other cases only part of the karyomastigont system is massively duplicated, resulting in mononucleate cells with huge numbers of flagella and complex cytoskeletons [4], [6], [7], [19], [20]. Each of these types evolved independently, and each has probably evolved more than once from distinct and simpler ancestors. However with our incomplete knowledge about the simpler forms, together with an overabundance of clades comprised entirely of “unidentified environmental sequences”, it is difficult to reconstruct these events.

Here we describe *Cthulhu macrofasciculumque* found in *Prorhinotermes simplex* (Hagan), and *Cthylla microfasciculumque*, found in *Reticulitermes virginicus* (Banks), newly described genera and type species of relatively small parabasalian termite symbionts that address both these issues. These two similar but morphologically distinct genera are the first identified members of a clade of unidentified environmental sequences derived from several distantly related rhinotermitids and kalotermitids. Altogether, these new genera help provide a morphological framework to a previously uncharacterized lineage of environmental sequences, and *Cthulhu* in particular represents a newly identified morphological type for Parabasalia, as well as a new case of accelerating size and complexity.

## Results and Discussion

### Barcode identification of termite hosts

**Figure 1:**
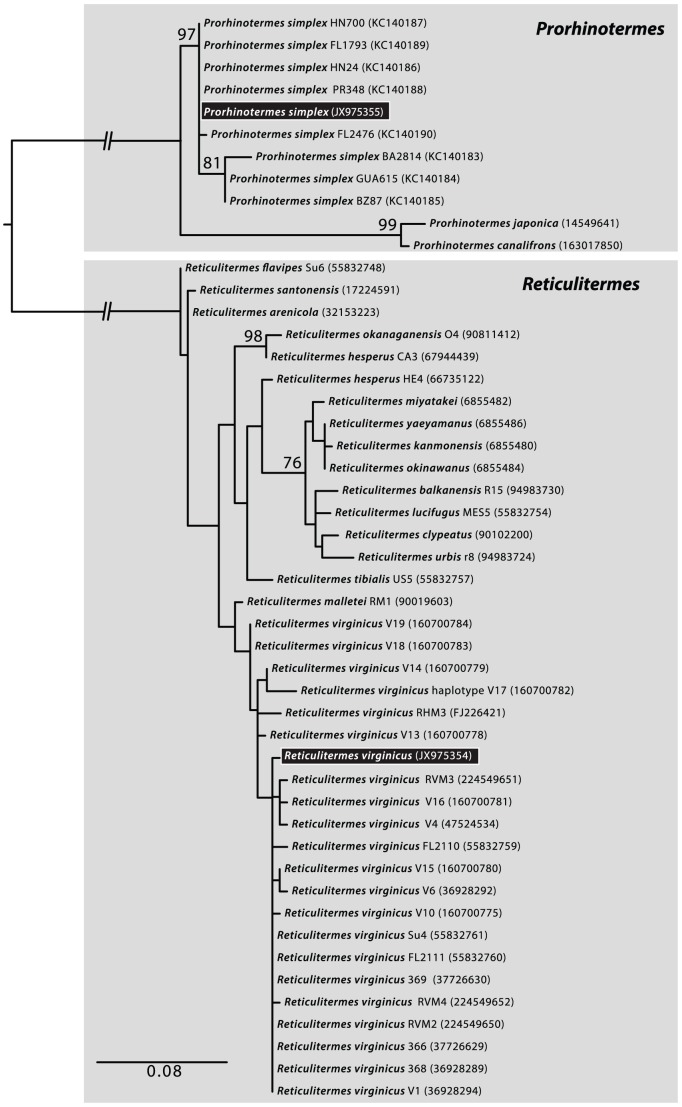
Barcode identification of termite hosts based on mitochondrial 16S (LSU) rRNA. At the top are all available barcodes from the genus *Prorhinotermes* and below are representative barcodes from all available species of *Reticulitermes* including represenative barcodes from *R. virginicus* (22 of 33 available sequences). Isolates used in this study are indicated by black boxes. Since no *P. simplex* barcodes were available, they were generated here from samples independently isolated from Florida, Puerto Rico, Bahamas, Belize, Guatemala, and Honduras.

The misidentification of host termites has often proved to be a complicating factor in the study of parabasalian symbionts. To avoid this issue, we first acquired a positive identification of all termite species by both examining their morphology and by characterizing mitochondrial 16S rRNA (mtLSU rRNA), an established DNA barcode marker for lower termites. Barcode sequences from field identified termites confirmed the identification of *R. virginicus* (Figure 1A), which shared 98.9% identity with a large number of *R. virginicus* barcodes (*R. virginicus* may be polyphyletic, but our isolate branches with the main group of *R. virginicus* barcodes). No comparable barcode sequence for *P. simplex* is available in public databases, so we also barcoded eight other isolates from across the Caribbean basin (including Central American records for *P. simplex* from Guatemala, Honduras, and Belize, all of which represent new ranges for this species). Comparing these and our *P. simplex* isolate confirmed its identification with a 99.7% shared identity. (Figure 1B). We also analyzed environmental sequences from three other termites, *Calcaritermes nearcticus* (Snyder), *Cryptotermes cylindroceps* (Scheffrahn & Krecek), and *Heterotermes tenuis* (Hagan), the identities of which were all confirmed by barcoding that was reported previously [21], [22].

### Morphology of Cthulhu macrofasciculumque

**Figure 2:**
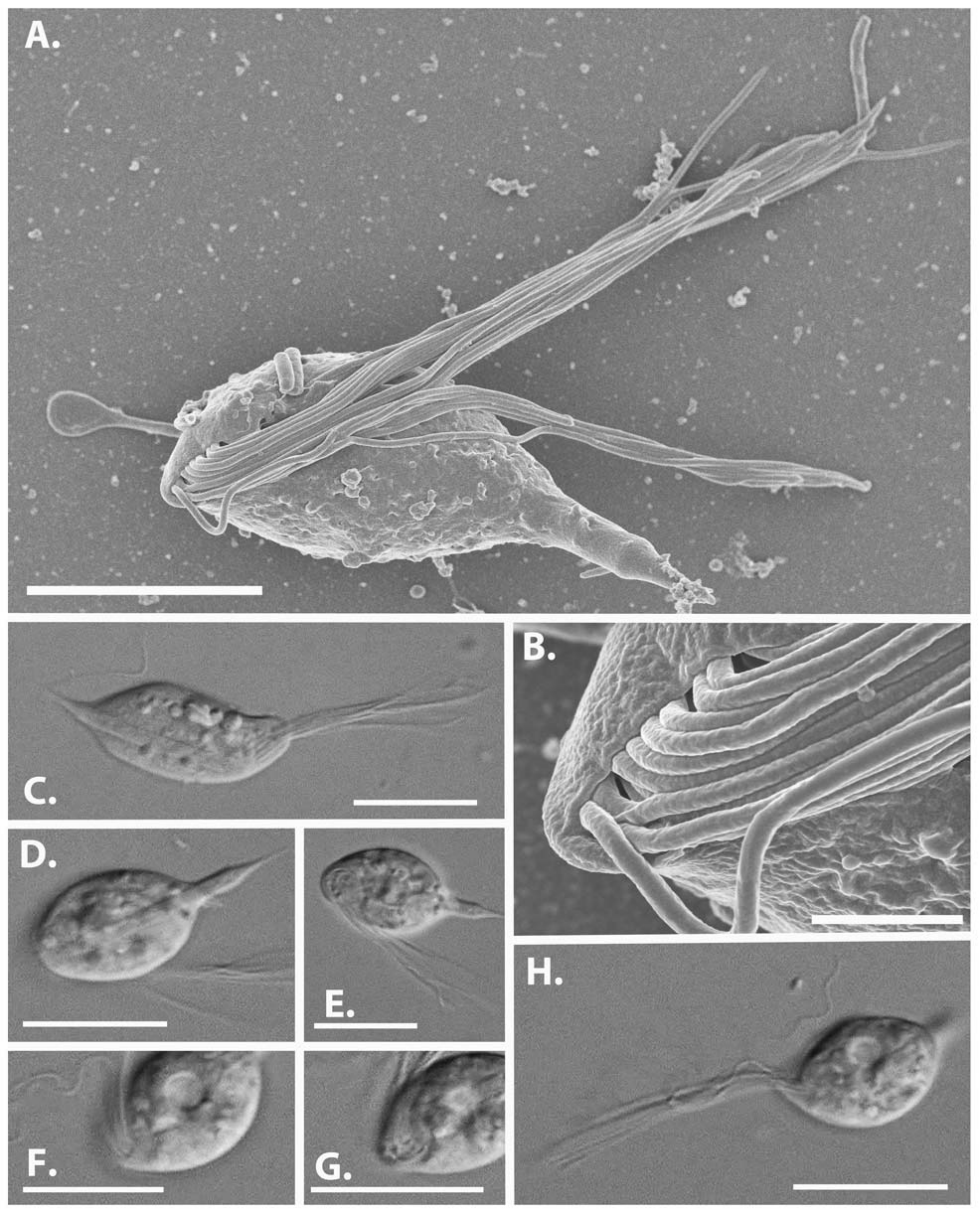
Morphology of *Cthulhu macrofasciculumque* by differential interference contrast light microscopy (LM) and scanning electron microscopy (SEM). (A) SEM showing overall characteristics of Cthulhu, including the posteriorly protruding axostyle and subanterior emergence of a bundle of flagella. Because they are bundled an exact number is hard to conclude, but 20 distinct flagella can be counted in this picture and in (B), which is a detailed view of the flagellar emergence. This flagellar bundle has an elongated shape, but others (e.g., G) appear rounded. (C & D) LM showing the overall body plan of the cell, including the pointed protruding axostyle which extends to the slightly subanterior flagellar emergence point where the single nucleus is situated (not shown), and the flagellar bundle. (E–H) Various views of the flagellar bundle and its emergence point in different cells and at different stages of the flagellar beat. It may appear as a single tight bundle projecting foraward (H), dissociated and trailing (D & E), and the emergence of the bundle may appear rounded (F & G) or band-like (E). In many views striations beneath the surface where the flagella emerge are visible (C & H). All scale bars are 10 µm except (B), which is 2 µm.

Observing the hindgut community of *P. simplex* revealed a number of large and conspicuous hypermastigonts (e.g., *Pseudotrichonympha* and *Holomastigotoides*), but we also noted an intermediate sized flagellate with interesting characteristics (Figure 2). Cells matching the same overall morphology were also observed in scanning electron microscopy of *P. simplex*. Cells were abundant in several host individuals and ranged in size from 17.8 to 23.3 µm in length and 6.7 to 11.4 in width (N = 13). A single anterior nucleus (which was difficult to observe in still cells but could be seen in moving cells more easily) was cupped within the curvature of a large and robust axostyle, that extended from the nucleus to the posterior end of the cell (generally protruding by almost 5 um), where it consistently terminated in an emergent point (Figure 2A, C, D). The cell surface is smooth, lacking any visible glycocalyx matrix, and not obviously associated with epibiotic bacteria (aside from a couple of possibly adhered bacteria in Figure 2A).

**Figure 3:**
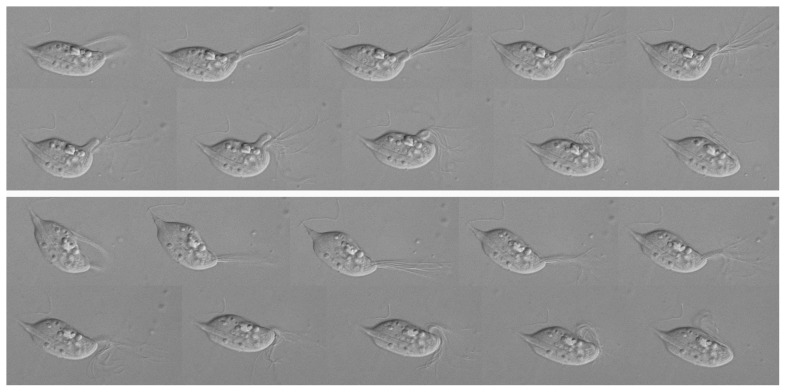
Two time series showing the flagellar beat pattern characteristic of *Cthulhu macrofasciculumque* both with a cytoplasmic undulation (top rows) and without (bottom rows). Stills are every second frame of video shot at 30 frames per second.

The anterior end of the cell was dominated by a large single tuft of flagella that beat with a complex synchronous pattern (Figure 3). The flagella bundled together and extended anteriorly as a single unit (Figure 2C, H, Figure 3), then as a unit thrust back forming a ‘hook’, typically unbundling in the backstroke. Due to the bundling and relatively large number of flagella, it was difficult to count flagella accurately in multiple individuals, but disrupted bundles appear to contain about 20 flagella, and 20 flagella could be counted in SEM images (Figure 2A). These flagella emerge slightly sub-anteriorly in a bundle that is often slot-shapped, but appears to be somewhat plastic (Figure 2A, B, G, Video S1). In some views, a single recurrent flagellum is also visible (Figure 2C, Video S1), but it is hard to distinguish a true recurrent flagellum from an unbundled anterior flagellum that is caught on the axostylar projection. Figure 3 shows two consecutive flagellar strokes, where it can be seen that the beat pattern is sometimes accompanied by a visible cytoplasmic undulation that starts at the flagellar emergence point and proceeds to the posterior of the cell (Figure 3: upper series), and sometimes not (Figure 3: lower series). These undulations do not resemble typical undulating membranes, since they appear to come from within the cell rather than from an adherent flagellum.

The parabasalian symbionts of *P. simplex* have been observed in several other studies [1], but only the larger hypermastigotes were noted, and no other parabasalian has been reported with the suite of morphological characteristics we observed in these cells. Based on this, and the results of molecular phylogenetic analysis from manually isolated cells (see below), we consider this to be a new genus and species, described above as *Cthulhu macrofasciculumque*.

### Morphology of Cthylla microfasciculumque

**Figure 4:**
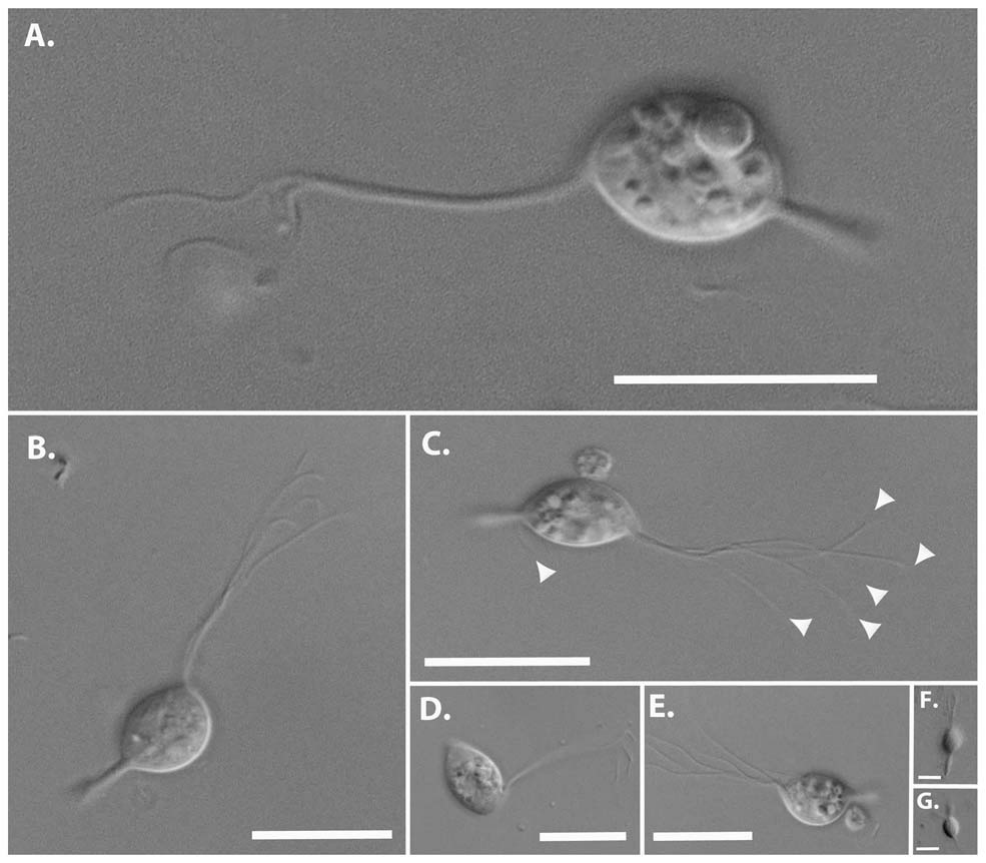
Morphology of *Cthylla microfasciculumque* by differential interference contrast light microscopy. (A) Overall body plan of Cthylla cells showing shape and size, a posteriorly protruding axostyle and subanterior emergence of flagellar bundle. Flagella typically bundle during the forward stroke of the flagellar beat but even then dissociate at the tips (A–C). In the backstroke, the bundle substantially dissociates (D–E). When dissociated, five anterior flagella can be seen consistently (B and clustered arrowheads in C), and in many views a single recurrent flagellum is also evident around the axostyle protrusion (A & lone arrowhead in C). (F &G). Low magnification of a small flagellate observed in *Heterotermes tenuis* with a small bundle of anterior flagella and a robust protruding axostyle similar to those of *Cthylla*. According to molecular surveys *H. tenuis* harbours the closest known relative of *Cthulhu*, and we predict that the environmental sequence is derived from this flagellate. All scale bars are 10 µm.

Several of the large and easily recognizable members of the *R. virginicus* hindgut community have been described previously, including *Trichonympha*, *Spirotrichonympha*, *Dinenympha*, and *Pyrsonympha* [1]. We observed all of these types, but also noted a small flagellate that shared some core characteristics with *Cthulhu* (Figure 4A–E). This flagellate also has a single anterior nucleus associated with a robust axostyle that projects at the posterior and terminates with a point (Figure 4A, B, C, Video S2). However, it is markedly smaller than *Cthu. macrofasciculumque*, being 10.4 to 15.0 µm in length and 6.6 to 9.6 µm in width (N = 14). It also bears only five anterior flagella (Figure 4C), and in many views also appears to have a single recurrent flagellum (Figure 4B, C, E). The flagella beat pattern was highly similar to that of *Cthu. macrofasciculumque*, consisting of a forward movement of bundled flagella (Figure 4A), which then hook and unbundle during a reverse thrust (Figure 4C, Video S2). The recurrent flagellum was not observed to be adhered to the body and no undulations were observed. Based on this unique set of characteristics, and its molecular phylogenetic position (see below), we consider this to be a distinct genus from *Cthulhu*, and due to its smaller size we named it *Cthylla*, who was the secret daughter of Cthulhu.

### Molecular phylogeny of cthulhumonads and hexamastigids

To determine the phylogenetic position of *Cthulhu* and *Cthylla*, we characterized the SSU rRNA from both new species. For *Cthulhu*, SSU rRNA was sequenced from 5 individual manually isolated cells and from two pools of 5 cells and a single pool of 12cells, and from whole *P. simplex* gut contents. Altogether 10 individual clones were sequenced from manually isolated cells, and found to share 99% identity. Two sequences from whole hindgut material were also found to share 99% identity with those from isolated cells, altogether suggesting the cells matching the morphology of *Cthu. macrofasciculumque* were a single coherent species. From *Cthylla*, SSU rRNA was sequenced from 7 individual manually isolated cells and from whole *R. virginicus* gut contents. Four sequences from whole hindgut material were also found to share 98% identity with those from isolated cells, altogether suggesting the cells matching the morphology of *Cthy. microfasciculumque* were also a single coherent species. We also sequenced SSU rRNA from whole gut contents of three other termites, *Cryptotermes cylindroceps*, *Calcaritermes nearcticus*, and *Heterotermes tenuis*, which yielded environmental sequences that were closely related to *Cthulhu* and *Cthylla*.

**Figure 5:**
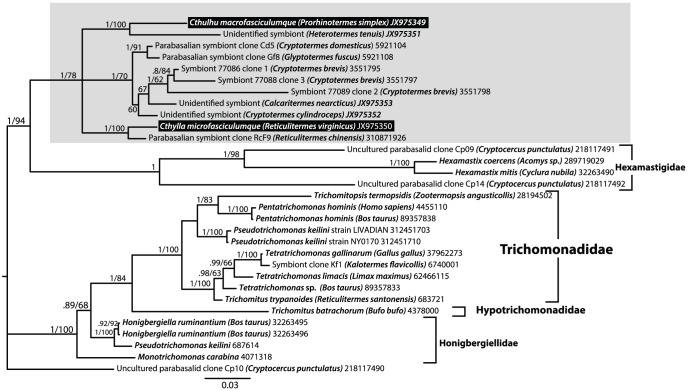
Phylogenetic relationships between *Cthulhu*, *Cthylla*, environmental sequences, and closely related parabasalians. Bayesian tree with posterior probabilities (upper) and maximum likelihood bootstraps (lower) indicated for each node, and major identified groups named to the right. *Cthulhu* and *Cthylla* are both related to unidentified environmental sequences from *Heterotermes tenuis* and *Reticulitermes chinensis*, respectively. They are all closely related to a large clade of unidentified environmental sequences from *Cryptotermes*, *Glyptotermes*, and *Calcaritermes*, which has previously been hypothesized to represent *Tricercomitus*. If true, then Cthulhu and Cthylla would be best considered members of the Tricercomitidae, thought this will depend on molecular characterization for this group.

In SSU rRNA phylogenies including a broad representation of parabasalian diversity, *Cthulhu* and *Cthylla* sequences branch together with the genus *Hexamastix* and a group of formerly unidentified environmental sequences from other termite hindguts (Figure S1). A detailed analysis of this subgroup of parabasalia and closely related trichomonads used as an outgroup (Figure 5), shows that *Cthulhu* and *Cthylla* belong (with high/moderate support) to a group of environmental sequences from *H. tenuis* (JX975351: this study) and *Reticulitermes chinensis* (310871926), respectively. This suggests flagellates with similar morphology may be found in these termites. We have neither species available to investigate, but we did review video footage from the same isolate of *H. tenuis* that our environmental sequence was derived, and identified two small flagellates from which the *Cthulhu*-related sequences might plausibly be derived (Figure 4F–G). Unfortunately the footage was targeted at larger hypermastigotes and was therefore at too low a magnification to make out details, but one can see 10 µm flagellates with a bundle of anterior flagella and a protruding axostyle.

*Cthulhu*, *Cthylla*, and their environmental relatives are in turn related to a cluster made up entirely of environmental sequences from a diverse group of kalotermitids (*Cryptotermes*, *Glyptotermes*, and *Calcaritermes*), and more distantly to the genus *Hexamastix* and several more unidentified sequences from termites, including some we identified from termite whole hindgut preparations: unidentified symbionts from *Cryptotermes cylindroceps* (JX975352) and *Cryptotermes nearcticus* (JX975353) (Figure 5). According to the recent classification of Čepička et al. (2010), this would place the cthulhumonads within the Honigbergiellida, which primarily includes the genera *Hexamastix* and *Tricercomitus* [9]. Interestingly, Čepička et al. singled out the clade of unidentified sequences that we now find are closely related to *Cthulhu* and *Cthylla* and hypothesize based on its distribution in termites and phylogenetic position that this lineage corresponds to the genus *Tricercomitus*. Both *Cthulhu* and *Cthylla* share certain features in common with the morphology of *Tricercomitus* and *Hexamastix*, which are about the same size as *Cthylla*, and also have robust, protruding axostyles ending in a point and lacking undulating membranes: [2]. *Hexamasitx* has five anterior flagella and a short recurrent one, like *Cthylla*, but *Tricercomitus* has only three anterior flagella and a very long recurrent one, unlike either *Cthulhu* or *Cthylla*. Taking morphology and phylogeny together, we speculate that both *Hexamastix* and *Tricercomitus* as currently described will emerge as polyphyletic genera in need of revision due to the difficulty in identifying and describing these small taxa without molecular data. If the unidentified clade related to *Cthulhu* and *Cthylla* does indeed turn out to correspond to the flagellates called *Tricercomitus* in the kalotermitids, then it will be important to characterise molecular data from *Tricercomitus termopsidis* (Kirby) in *Zootermopsis angusticollis* (Hagan) since this is the type host for the type species [23], and it is important to eventually test these hypotheses with additional genes, which are presently too sparsely sampled to allow such tests.

### Taxonomic Summary

**Assignment**. Eukaryota; Parabasalia.

***Cthulhu*** **n. g. James and Keeling, 2012.**

urn:lsid:zoobank.org:act: 25BCAD11-B42E-4105-826C-0459AE4EC5A8

**Type species**: *Cthulhu macrofasciculumque* (Figure 2C, Video S1)

**Type host**: *Prorhinotermes simplex* (Isoptera, Rhinotermitidae: barcode JX975355)

**Description**: Small parabasalian flagellate with a single anterior nucleus associated with a robust axostyle. Axostyle extends the entire length of the cell, and projects at the posterior where it terminates in a point. Approximately 20 flagella emerge in a single slightly sub-anterior bundle. Anterior flagella beat coordinately, forming a single large bundle that rolls anteriorly to the fully extended position and then dissociates into loose flagella that whip posteriorly. Possibly also a single recurrent flagellum.

**Etymology**: The name is based on the fictional many tentacled, cephalopod-headed demon found in the writings of H. P. Lovecraft, specifically The Call of Cthulhu. The tentacle-headed appearance given by the coordinated beat pattern of the anterior flagellar bundle of *Cthulhu cells* is reminiscent of this demon. The name is supposedly impossible to pronounce as it comes from an alien language, but currently it is most often pronounced “ke-thoo-loo”.

**Cthulhu macrofasciculumque n. sp. James and Keeling, 2012**

urn:lsid:zoobank.org:act:B5FDCF7C-386D-4793-8EB0-61F4B0063BFA

**Type host**: *Prorhinotermes simplex* (Isoptera, Rhinotermitidae: barcode JX975355)

**Type locality**: Ft. Lauderdale, Secret Woods County Park, Florida, USA: lat. 26.08567, long. −80.18017.

**Host collection**: University of Florida termite collection, accession number FL1563. Collector B. Maharajh. Collected Sept. 15, 2002.

**Description**: Parabasalian flagellate with morphological characters for the genus *Cthulhu*. Cells are 17 to 24 µm in length and 7 to 12 µm in width and have a minimum of 20 anterior flagella. Found in the hindgut of *Prorhinotermes simplex*. Distinct SSU rRNA sequence (GenBank accession number JX975349).

**Hapantotype**: Microscope slide deposited at the Beaty Biodiversity Museum, University of British Columbia, Vancouver, Canada under accession number MI-PR200.

**Gene sequence**: SSU rRNA accession number JX975349.

**Etymology**: Species name refers to the large (macro) bunch (fasciculumque) of flagella.

**Cthylla n. g. James and Keeling, 2012.**

urn:lsid:zoobank.org:act 0C373970-AD9F-4A3A-930B-36D82FC8683C

**Type species**: *Cthylla microfasciculumque* (Figure 4A, Video S2)

**Type host**: *Reticulitermes virginicus* (Isoptera, Rhinotermitidae: barcode JX975354)

**Description**: Parabasalian flagellate with a single anterior nucleus associated with a robust axostyle. Axostyle extends the entire length of the cell, and projects at the posterior where it terminates in a point. Five flagella emerge in a single slightly sub-anterior bundle. Anterior flagella beat coordinately, forming a single large bundle that rolls anteriorly to the fully extended position and then dissociates to individual flagella that whip posteriorly. Possibly also a single recurrent flagellum.

**Etymology**: The name is based on the fictional Cthylla (often pronounced ke-thil-a), who was the secret daughter of Cthulhu in later writing inspired by H. P. Lovecraft. Though never described, Cthylla is generally portrayed as a winged cephalophod. It is here named as a smaller and simpler relative of the parabasalian genus *Cthulhu*.

**Cthylla microfasciculumque n. sp. James and Keeling, 2012**

urn:lsid:zoobank.org:act:C5109A93-A1EC-4362-AEBF-D2E538C55D66

**Type host**: *Reticulitermes virginicus* (Isoptera, Rhinotermitidae: barcode JX975354)

**Type locality**: Ft. Lauderdale, Secrete Woods County Park, Florida, USA: lat. 26.08567, long. −80.18017.

**Host collection**: University of Florida termite collection, accession number FL2261. Collector R. H. Scheffrahn. Collected Feb 21, 2005.

**Description**: Parabasalian flagellate with morphological characteristics of the genus *Cthylla*. Cells are 10 to 15 µm in length and 6 to 10 µm in width. Found in the hindgut of *Reticulitermes virginicus*. Distinct SSU rRNA sequence (GenBank accession number JX975350)

**Hapantotype**: Microscope slide deposited at the Beaty Biodiversity Museum, University of British Columbia, Vancouver, Canada under accession number MI-PR201.

**Gene sequence**: SSU rRNA accession number JX975350.

**Etymology**: Species name refers to the small (micro) bunch (fasciculumque) of flagella.

## Materials and Methods

### Host identification and barcoding

*Paraneotermes simplex* was collected Sept. 15, 2002 in Secret Woods County Park, Ft. Lauderdale, Florida, (lat. 26.08567, long. −80.18017) and deposited in the University of Florida termite collection, accession number FL1563. *Reticulitermes virginicus* was collected on Feb 21, 2005 at the same location and deposited in the University of Florida termite collection, accession number FL2261. *Cryptotermes cylindroceps*, *Calcaritermes nearcticus*, and *Heterotermes tenuis* were collected as reported previously [21], [22]. No specific permits were required for the described field studies, the locations are not privately owned or protected, and no endangered or protected species were collected.

DNA barcodes at the mitochondrial 16S (LSU) rRNA marker for *P. simplex* and *R. virginicus* were amplified using LR-N-13398 CGCCTGTTTATCAAAAACAT and LR-J-13007 TTACGCTGTTATCCCTAA under conditions previously described [21], [22]. Since no comparable *P. simplex* barcode was available, we also barcoded *P. simplex* from eight other independent isolations (University of Florida Termite collection accessions FL2476, FL1793, PR348, BA2814, BZ87, GUA615, HN24, and HN700) from Florida, Puerto Rico, Bahamas, Belize, Guatemala, and Honduras (the latter three being new regional and country records for this species). These new barcodes were submitted to GenBank under accessions KC140183-90. These were aligned with all available *Paraneotermes* and *Reticulitermes* mt LSU termite barcodes from Genbank using MAFFT [24], and refined by eye using SeaView [25]. Poorly aligned regions were automatically removed with trimAl using a gap threshold of 0.9 [26]. AIC weight as calculated with the perl script MrAIC.pl [27] was used to determine the evolutionary model that best fit the data, which corresponded to GTR+Γ+I in all cases. For the termite phylogeny, Maximum Likelihood (ML) estimation was carried out using PhyML 3.0 [28] with statistical support inferred from 1000 bootstrap replicates.

### Microscopy

Whole termite guts were dissected in Trager medium U [29]. Light microscopy was performed on living cells using a Zeiss Axioplan 2 compound microscope using differential interference contrast (DIC) optics. Image capture was carried out using a Canon XL-M1S, from which stills were captured and HD video archived.

For scanning electron microscopy (SEM), whole hindgut contents were mounted on a poly-L-lysin coated coverslip, then fixed in 2.5% glutaraldehyde (v/v; final concentration) diluted in Trager's medium U overnight at 4°C. Samples were dehydrated using a graded ethanol series and critical point dried in a Tousimis Sandri 795 CPD (Rockville, MD, USA). The dried coverslips were mounted onto aluminum stubs and sputter-coated with 5-nm thick gold (Cressington High Resolution Sputter Coater, Cressington Scientific Instruments, Ltd., Watford, UK). The scanning electron microscopy was performed using a Hitachi S4700 (Hitachi, Japan).

#### Molecular and phylogenetic analyses

Target cells were isolated by micropipette using a Zeiss Axiovert 2 microscope and each isolated cell was photographed using a QImaging MicroImager II camera (data not shown). DNA was isolated using the Epicentre Masterpure Complete DNA and RNA purification Kit (Madison, WI). SSU rRNA gene sequences were amplified from single cells, small pools of identical cells, and whole hindgut contents using eukaryote-specific primers 5′-TGC GCT ACC TGG TTG ATC CTG CC-3′ and 5′-TGA TCC TTC TGC AGG TTC ACC TAC-3′ as described previously [21], [22]. PCR products were separated by agarose gel electrophoresis, cloned using the StrataClone PCR Cloning Kit (Stratagene, Mississauga, ON), and sequenced on both strands using BigDye Terminator v 3.1. For each new taxon, a representative SSU rRNA gene either identical to or closest to the consensus was chosen to represent the species in phylogenetic analyses and submission to GenBank.

Parabasalian and host rRNA phylogenies were inferred using ML and Bayesian tree reconstruction methods, with PHyML v.3 [28] and MrBayes v.3.2 [30], respectively. The evolutionary model that best fit the data was determined with mrAIC.pl (Nylander, J. A. A. 2004. MrAIC.pl. Program distributed by the author. Evolutionary Biology Centre, Uppsala University), and corresponded to GTR+Γ+I in both cases. For PHyML, eight rate categories were used with the gamma shape parameter and the proportion of invariable sites estimated from the data. The SPR method of tree improvement was chosen, and 1000 bootstrap replicates performed for evaluating the support. For MrBayes, the inference used four Metropolis-coupled Markov Chain Monte Carlo consisting of 1,000,000 generations with sampling every 100 generations. The average standard deviation of split frequencies was used to assess the convergence of the two runs. Bayesian posterior probabilities were calculated from the majority rule consensus of the tree sampled after the initial burnin period corresponding to 20% of the generations (200,000 generations).

## Supporting Information

Video S1 **Movie of live** ***Cthulhu macrofasciculumque*** **showing overall body plan and the flagellar beat pattern with and without cytoplasmic undulation.**
(MP4)

Video S2 **Movie of live** ***Cthylla microfasciculumque*** **showing overall body plan and the flagellar beat pattern.**
(MP4)

Figure S1 **Maximum likelihood tree with a broad representation of parabasalian diversity, inferred with RAxML and the GTR+gamma model of evolution.** New sequences from Cthulhu and Cthylla are shown white on black.
(EPS)
